# Association of 152 Biomarker Reference Intervals with All-Cause Mortality in Participants of a General United States Survey from 1999 to 2010

**DOI:** 10.1093/clinchem/hvaa271

**Published:** 2021-03-01

**Authors:** Nam Pho, Arjun K. Manrai, John T. Leppert, Glenn M. Chertow, John P.A. Ioannidis, Chirag J. Patel

**Affiliations:** a Department of Biomedical Informatics, Harvard Medical School, Boston, MA; b Department of Medicine, Stanford University School of Medicine, Stanford, CA; c Department of Urology, Stanford University School of Medicine, Stanford, CA; d Department of Epidemiology and Population Health, Stanford University School of Medicine, Stanford, CA; e Department of Biomedical Data Science, Stanford University School of Medicine, Stanford, CA; f Department of Statistics, Stanford University School of Humanities and Sciences, Stanford, CA.

## Abstract

**BACKGROUND:**

Physicians sometimes consider whether or not to perform diagnostic testing in healthy people, but it is unknown whether nonextreme values of diagnostic tests typically encountered in such populations have any predictive ability, in particular for risk of death. The goal of this study was to quantify the associations among population reference intervals of 152 common biomarkers with all-cause mortality in a representative, nondiseased sample of adults in the United States.

**METHODS:**

The study used an observational cohort derived from the National Health and Nutrition Examination Survey (NHANES), a representative sample of the United States population consisting of 6 survey waves from 1999 to 2010 with linked mortality data (unweighted *N*=30 651) and a median followup of 6.1 years. We deployed an X-wide association study (XWAS) approach to systematically perform association testing of 152 diagnostic tests with all-cause mortality.

**RESULTS:**

After controlling for multiple hypotheses, we found that the values within reference intervals (10–90th percentiles) of 20 common biomarkers used as diagnostic tests or clinical measures were associated with all-cause mortality, including serum albumin, red cell distribution width, serum alkaline phosphatase, and others after adjusting for age (linear and quadratic terms), sex, race, income, chronic illness, and prior-year healthcare utilization. All biomarkers combined, however, explained only an additional 0.8% of the variance of mortality risk. We found modest year-to-year changes, or changes in association from survey wave to survey wave from 1999 to 2010 in the association sizes of biomarkers.

**CONCLUSIONS:**

Reference and nonoutlying variation in common biomarkers are consistently associated with mortality risk in the US population, but their additive contribution in explaining mortality risk is minor.

## Introduction

While medical practitioners use a spectrum of physiological and biomarker measurements (e.g., serum glucose, serum cholesterol, blood pressure), little is known with respect to whether “normal” variation of common biomarkers is associated with death in a nondiseased, healthy population (1, 2). Diagnostic testing is often reported with a reference interval to provide context in interpreting measurement results of a biomarker (3). For several biomarkers, e.g., reflecting kidney or liver function, extreme values, outside the reference interval, are clearly associated with the risk of death; however, when outlying and clearly abnormal values are excluded, the association of the more modest, largely “normal” biomarker variation with mortality remains elusive. Sometimes physicians consider whether to perform diagnostic testing using biomarkers in largely healthy people, but then one is stuck with interpreting whether nonoutlier results confer any predictive information. Moreover, depending on their subspecialty, practitioners may focus on one or a few biomarkers at a time (4).

There are few data-driven approaches that search for multiple variables of diverse organ systems including the kidney, bone, and liver, that might be associated with mortality in a general and otherwise healthy population (5, 6). Furthermore, investigations that utilize administrative data (e.g., electronic health records, insurance claims) may be fraught with selection bias (e.g., administrative samples may have a higher prevalence of unhealthy individuals than noninstitutionalized populations) (7–9). Over the past few decades, the challenges of “over testing” and screening in specific use-cases have rightly been considered (10–13). For example, Reed and colleagues report the number of tests that lead to false positives that is based on statistical theory. Others, such as Durbridge et al., consider the role of defining critical values of diagnostic tests on admission to the hospital in a biased and nonrepresentative sample. These are important examples; however, they need updating in the face of the potential battery of clinical tests available across a diverse segment of the US population. It would be useful to know whether common laboratory tests of biomarkers might be predictive of mortality risk in an unselected, general population without obvious disease. While we focused on mortality as it is an endpoint all humans face, these biomarkers are often interpreted to stage disease risk and therapeutic decision-making, such as cholesterol and heart disease risk. On the other hand, biomarker values for a patient in critical condition are, and should be, interpreted entirely differently.

To counter selective testing and reporting we have employed methods for systematic association studies, specifically the X-wide association study (XWAS) (14, 15), aiming to search for and validate biomarkers associated with disease and disease-related biomarkers (i.e., mortality) (16–19).

Previous studies have explored similar questions drawing from EHR/EMR data (12). However, we leverage XWAS methods to systematically assess 152 biomarkers associated with all-cause mortality in a noninstitutionalized participant population from the US Centers for Disease Control and Prevention National Health and Nutrition Examination Survey (NHANES) 1999–2000, 2001–2002, 2003–2004, 2005–2006, 2007–2008, and 2009–2010 surveys (20–25). There exist selective biases from using EHR/EMR data; the process of a diagnostic test being ordered is an indication of mortality when extracting observations about patients from a medical system (26). We define biomarkers broadly to include any indicator of a particular disease state or some other physiological state of an organism such as body size, cardiorespiratory vital signs, lifestyle factors, and laboratory markers of metabolism, inflammation, liver, and kidney function. These biomarkers are often used in the clinic for diagnostic testing or general health and wellness monitoring. We associated each of these 152 biomarkers with mortality using Cox proportional hazards regression. Second, we replicate findings by seeking concordant associations in a minimum of 3 independent NHANES surveys and estimated the survey-to-survey variability of the associations. Finally, we determined the risk for death of individuals who were healthy by different criteria, including those who (*a*) were within population reference intervals of the biomarker, (*b*) did not have self-reported history of chronic disease (i.e., heart disease, stroke, cancer, type 2 diabetes, obesity, and arthritis), or (*c*) did not seek healthcare in the year prior to the survey.

## Materials and Methods

### NATIONAL HEALTH AND NUTRITION EXAMINATION SURVEY

The NHANES is a survey executed by the Centers for Disease Control and Prevention (CDC) and the National Centers for Health Statistics (NCHS) to monitor the health of a representative population of the United States every 2 years and available to the public (27).

Participants of the NHANES are selected using a multistage probability sampling design (28). The CDC and NCHS collected survey information through in-person interviews and various testing, including bodily measures, biomarkers, and physiological indicators, in a comprehensive physical examination at mobile examination centers. Protocol approval and written informed consent was obtained by the NCHS Institutional Review Board for participants 18+ years of age. We conducted our study in accordance with the Strengthening the Reporting of Observational Studies in Epidemiology (STROBE) guidelines (29).

### CLINICAL AND DIAGNOSTIC TESTS

The NHANES contained 339 unique biomarkers measured in participants in 6 surveys from 1999 to 2010. In our investigation, we added the additional criterion of being present across at least 3 survey years to enhance replication, resulting in 152 total biomarkers (Table 1 for examples; Table 1 in the online Data Supplement for a list of all) considered in this study. All of these biomarkers may be employed for clinical diagnostic or general health and wellness monitoring purposes in different settings.

We grouped the biomarkers into different categories. The total number of biomarkers per category included 15 blood parameters (e.g., red blood cell count), 4 on blood pressure and heart rate related measures (e.g., systolic and diastolic blood pressure), 11 on body measures and adiposity (e.g., BMI), 41 on bone density measures, 3 on cancer diagnostics (e.g., prostate specific antigen), 8 for cardiovascular disease indicators (e.g., total cholesterol), 13 on kidney function (e.g., serum creatinine), 8 on liver function (e.g., alanine aminotransferase), 6 on metabolic-related function (e.g., glucose), 1 nutritional indicator, 14 indicators on physical fitness, and 16 on physical and mental functioning.

### PRIOR HEALTHCARE UTILIZATION AND BIOMARKER REFERENCE INTERVALS

We hypothesized that participants’ recent healthcare utilization might influence the association of biomarker and mortality. Therefore, we associated 2 indicators of healthcare utilization, the number of times the participant had received healthcare and whether the individual required overnight hospitalization 1 year prior to being surveyed, with each of the biomarkers in each survey year separately using a survival regression model. Other adjusting covariates included age, age-squared, sex, race, and income-to-poverty ratio for each independent survey separately (e.g., 1999–2000). Race was self-reported and categorized as “Nonhispanic White,” “Nonhispanic Black,” or “Mexican American.” The income-to-poverty ratio was calculated by dividing self-reported household income by the survey year’s Department of Health and Human Services poverty guideline determined by financial eligibility for certain federal programs (e.g., Head Start, Supplemental Nutrition Assistance Program, Special Supplemental Nutrition Program for Women, Infants, and Children, the National School Lunch Program). We combined association sizes for each of the surveys for the healthcare utilization variables using a random effects meta-analytic method and with a restricted maximum-likelihood estimator of heterogeneity (30).

### SYSTEMATIC ASSOCIATIONS BETWEEN BIOMARKER REFERENCE INTERVALS AND MORTALITY

We systematically analyzed the association of each of the 152 biomarkers (Table 1, Supplemental Table 1) with mortality independently within each of the 6 separate surveys. We used survey-weighted Cox proportional hazards regression to associate the scaled value of the biomarker with mortality adjusted for age, age-squared, sex, race, income, the number of times the participant visited the hospital in the last year, and the number of times a participant sought healthcare (31). All selected biomarkers are continuous measurements.

Having a measurement within the top or bottom extrema of what is expected for a biomarker reference interval may be indicative of disease. To ensure that we considered results of diagnostic tests found within population reference intervals, we removed from analysis participants whose diagnostic tests were in population extrema, outside the interval covering the 10th to the 90th percentiles; i.e., the analysis focused on diagnostic test results within the 10th to 90th percentiles of population reference intervals of the initial test distributions. We empirically determined our cutoff selection with a sensitivity analysis in the form of a Bland–Altman plot to see how varying cutoffs influences the association sizes. To check the sensitivity of the associations to varying percentile cutoffs, we also conducted our systematic associations in the 10th (0.10, 0.90), 20th (0.20, 0.80), and 30th (0.30, 0.70) percentiles of the distribution and visualized the differences in association sizes in a Bland–Altman plot (Supplemental Fig. 1).

To facilitate comparison of association sizes between the 152 biomarkers, we scaled (mean-subtracted and divided by the standard deviation) the reported values. Therefore, association sizes reflect a 1 standard deviation (SD) increase in the distribution of the biomarker. We adjusted for multiple hypotheses with the false discovery rate (FDR) using a FDR <0.05 filter throughout the study. We report an overall hazard ratio (HR) or association size and significance calculated across all survey years available for a biomarker using a random effects meta-analysis technique with a restricted maximum-likelihood estimator of heterogeneity (30).

## Results

### STUDY DEMOGRAPHICS

Over 6 NHANES cohort years from 1999 to 2010 there was a total of 35 327 participants (43%) who fit our study criteria with a median time to followup of 6.08 years, yielding a total exposure of 210 697.4 person years (Table 2). The entire NHANES from 1999 to 2010 comprises 82 091 survey participants, consenting male and female participants from newborns to individuals over 80 years of age.

Associations among demographic characteristics and mortality (with indicated adjusted HR) are reported in Table 3. Older age was associated with mortality risk [HR = 3.82 (3.07, 4.75)] for a SD increase (25 years) in age. Women experienced a 39% lower risk for death relative to men [HR = 0.61 (0.57, 0.66)]. Increasing income-to-poverty ratio was associated with decreased risk for death [HR = 0.76 (0.73, 0.79)]. In 2010, the federal poverty level for an individual was determined to be $11 139 per year (32) or being at an income-to-poverty level of 1, each SD increase of $17 747.69 in annual income would decrease your risk of death by 24%. Nonhispanic African Americans [HR = 1.20 (1.07, 1.33)] had a higher risk for death than nonhispanic Whites. Last, the number of times an individual seeks healthcare from between 4 and 9 visits up from the national average of 2 to 3 [HR = 1.24 (1.19, 1.30)] and an overnight hospital stay [HR = 1.12 (1.09, 1.1)] within the past year were both associated with higher risk of mortality.

### BIOMARKERS ASSOCIATED WITH PRIOR-YEAR HEALTHCARE UTILIZATION AND HOSPITAL VISIT

We hypothesized that healthcare utilization prior to survey was associated with biomarker value and risk for mortality. We associated each of the 152 biomarkers iteratively with prior-year healthcare utilization, adjusting for age, age-squared, sex, race, and income in each of the surveys. We found 83 out of 152 biomarkers (54%) were associated with the number of times the participant received healthcare in the year prior to the survey (FDR < 0.05). Second, we found 64 out of the 152 biomarkers (42%) were associated with the number of times a participant visited the hospital in the prior year (FDR < 0.05). The median absolute value association size between the number of times a participant received healthcare and the 152 biomarkers was 0.4% [interpreted as percentage change in 1 SD of the biomarker per each additional healthcare visit CI: (0.14, 1.1)]. The median absolute value association size between the number of times a participant visited the hospital was 1.0% [CI: (0.3, 2.4)].

### RELATIONSHIP BETWEEN BIOMARKERS AND ALL-CAUSE MORTALITY

A heatmap of pairwise correlations between each clinical test shows modest global correlation (mean Pearson ρ = 0.1) with the exception of skeletal measures that are more positively correlated (mean Pearson ρ = 0.5) (Supplemental Fig. 2). We associated each of the biomarkers with all-cause mortality iteratively, adjusting for the demographic groupings of age, sex, race, income-to-poverty ratio, number of times an individual utilized healthcare, and whether the individual had an overnight hospitalization in the year prior to survey.

In our overall population analyses of the NHANES cohorts we found 20 (13% out of 152) biomarkers that were replicated with an FDR lower than 0.05 in at least one survey (Table 4). Liver, kidney, and blood markers including albumin [serum HR = 0.80 (0.76, 0.85) and urine HR = 1.24 (1.16, 1.32)] and alkaline phosphatase [HR = 1.23 (1.09, 1.39)] are notable inclusions. General, nonspecific stress markers such as C-reactive protein [HR = 1.19 (1.09, 1.30)] and those pertaining to the immune system including leukocyte (white blood cell, WBC) count [HR = 1.13 (1.06, 1.20)] and segmented neutrophil (polymorphonuclear leukocyte) number [HR = 1.18 (1.11, 1.25)] are also present. Multiple indicators of bone health were associated with mortality, including bone density measures of the thigh (e.g., trochanter, femur), hip (e.g., intertrochanter), lower-back (e.g., lumbar-pelvic), and neck. An increase in 1 SD of each bone measure represented at least a 20% decrease in mortality risk (HR < 0.8). We were able to explain 0.148 of variation (Nagelkerke *R*^2^) across all diagnostic tests indicative of models in addition to adjusting for the demographic groupings of age, sex, race, and income-to-poverty ratio compared to a null model with a *R*^2^ = 0.140 and only adjusting for the demographic variables.

### CORRELATION BETWEEN BIOMARKERS WITH REPLICATED ASSOCIATIONS

To assess the independent contribution of the identified biomarkers, we estimated their pairwise correlations. Among the biomarkers with replicated associations the mean Pearson pairwise correlation was modest [Pearson ρ = 0.10 (0.01, 0.29), Supplemental Fig. 3] and comparable to the full set of 152 explored biomarkers with mean 0.11 [0.01, 0.36] (Supplemental Fig. 2). The strongest diagnostic variables were largely independent of one another.

## Discussion

In this study, we associated values within the 10th and 90th percentile of values for 152 quantitative biomarkers often used in general health and wellness monitoring, disease risk assessment, clinical diagnosis, and medical decision-making with all-cause mortality. The cutoffs were selected after systematically performing a sensitivity analysis (Bland–Altman plot, Supplemental Fig. 1). We have demonstrated that the 10th and 90th percentile cutoffs were no different versus more stringent cutoffs at 20th and 80th percentiles or even 30th and 70th percentiles. We found 20 biomarkers (Table 4) within these intervals for liver, bone, and kidney dysfunction, such as albumin, C-reactive protein, and alkaline phosphatase were associated with mortality. The ranges of *I*^2^ values for each biomarker, which quantify the variation of HR between survey cohort years, are low (<1%) with the exception being alkaline phosphatase at 80.4%. Variation in alkaline phosphatase may be driven by hormonal state (e.g., puberty, menopause) or morphometric parameters (e.g., height, body weight) (33) but it is unclear whether the secular changes in these factors are driving the variation in HR between survey years. The final “panel” of biomarkers reflect major, known risk factor with associations to mortality consistent across demographic (i.e., sex, age, income, race) or medical utilization or perception (i.e., those with chronic medical conditions or increased healthcare utilization). Additional testing (up to all 20 biomarkers together) explained less than 1% of the overall variance in mortality risk (10, 13). In clinical contexts, we expect single abnormal biomarker values will explain more of the variation of specific clinical outcomes and trajectories. There are many burdensome diseases (e.g., cardiovascular disease and cancer), but biomarkers to assess future risk are only available for a handful of them or are designed for specific populations [e.g., Framingham Risk Score (34)]. Our study considers systematically all biomarkers associated with mortality among a representative sampling of noninstitutionalized individuals in the US. It is unknown how much “normal” biomarker variation is associated with mortality. Even the definition of population reference intervals—intervals that define a majority of the population as “normal” or “healthy” individuals—remains elusive. Further still, biomarkers used in clinical diagnostic testing may vary substantially between different demographics, including in children (35), as well as racial (36) or ethnic groups (37), and socio-economic strata (38–41). We focused on quantifying the risk for mortality across an array of biomarkers and found that, while reproducible across independent survey waves, the individual risk between biomarker values and death in otherwise healthy individuals was modest (42, 43).

Over testing is a risk due to the rise in the “incidentalome” (44) from precision medicine and the increasing practice of defensive medicine (45). The phenomenon of false positive findings due to multiple testing has been evaluated in the context of determining abnormal laboratory results (outside 95% limits). For example, the false positive rate increases with multiple testing, reaching 50% at 14 tests and 90% at 50 tests (13). Conversely, reports of risk for mortality for biomarker values whose values lie within the 10th to 90th percentile of the distribution have a large chance for being false positive. Aside from incidental findings and spurious results from the biomarker panel variation represented by the differing presentation and clinician diagnosis or treatment of the same conditions among patients, over testing results in increased cost of health-care as well as lost time before treatment among diseases where early intervention would be critical to a favorable outcome. In our investigation, we found statistically robust associations between several biomarkers and mortality; however, these diagnostic tests in concert only explained 0.8% of the variance of mortality risk (after taking into account age, sex, ethnicity, income, and healthcare utilization). Therefore, a large panel of these biomarkers may provide marginal information and thus their use would have unclear medical benefit or justification.

Despite the intention of the CDC to capture a representative US population, biomarker data in NHANES does not capture individual-level trends or trajectories. Study limitations also include missing data and an inconsistent picture of true demographic diversity (beyond ethnically Black or White individuals) across the US over time. While we sought to model reference intervals linearly by subsetting data points outside a given percentile, it may be more physiologically accurate to capture nonlinear relationships between biomarkers and mortality. Finally, while we focused on mortality because of it is of maximal clinical importance, diseases are also important to predict. Some tests may be better at prediction of disease versus mortality. In general, massive testing of healthy individuals should be approached with caution so that its results bring clinical utility in assessing risk of mortality or other diseases.

With the rise of personal health tracking technologies (e.g., smart watches, home sensors, fitness tokens) enabling continuous physiological monitoring and proliferation of direct to consumer services, it is becoming important to know which objective measures of health are most predictive of mortality and the onset of chronic conditions. These measures may merit prioritization for further clinical exploration. For example, abnormal results from red cell distribution width monitoring could be indicative of anemia (of chronic disease) or underlying autoimmune, chronic kidney disease, and even cancer. Nevertheless, even with high predictive ability, clinical benefit cannot be guaranteed (e.g., if the tests reflect conditions that are not modifiable or effective interventions do not exist). Our systematic approach may be extended to similarly evaluate the predictive yield also for new types of diagnostic measuring and monitoring offered by the advent of new technologies.

## Supplementary Material

Supplementary Information

Fig S3

Fig S1

Fig S2

## Figures and Tables

**Table 1.** Number and examples of clinical and diagnostic variables selected for systematic analysis in association with mortality (152 total).

Clinical category	*n*	Examples
blood	15	Hematocrit (%)
		Hemoglobin (g / dL)
		Red blood cell count (million cells / μL)
blood pressure	4	60 second pulse
		Mean systolic
body measures	11	Body mass index (kg m^2^)
		Weight (kg)
		Waist circumference (cm)
bone	41	Lumbarspine BMD (g / cm^2^)
		Bone alkaline phosphatase (μg / L)
cancer	3	Free prostate specific antigen (ng / mL)
heart	8	Total cholesterol (mg / dL)
		Triglycerides (mg / dL)
		Homocysteine (μmol / L)
immune	12	Lymphocyte percentage (%)
		C-reactive protein (mg / dL)
		Monocyte (%)
kidney	13	Creatinine (mg / dL)
		Urine albumin (μg / mL)
		Sodium (mmol / L)
liver	8	Alkaline phosphatase (U / L)
		Aspartate aminotransferase (AST, U / L)
		Alanine aminotransferase (AST, U / L)
metabolic	6	C-peptide (nmol / L)
		Serum glucose (mg / d ^−1^)
		Insulin (μU / mL)
nutrition	1	Methylmalonic acid (μmol / L)
physical fitness	14	VO_2_ Max(mL / kg / min)
physical functioning	16	Condition 1 Trial 1 Failure Time (seconds)

**Table 2.** Demographic distribution in 6 NHANES cohort years 1999–2010 with linked mortality information.

	1999–2010	1999–2000	2001–2002	2003–2004	2005–2006	2007–2008	2009–2010
*n*	35 327	5443	5985	5610	5560	6219	6510
Age (SD)	47.4 (20.1)	47.2 (20.7)	46.9 (20.8)	47.5 (21.1)	45.2 (20.3)	49.3 (18.8)	48.2 (18.7)
Male (%)	48.1	46.8	47.5	48.0	48.1	49.3	48.7
Mortality (%)	10.4	19.3	16.1	13	7.7	5.7	2.1
Poverty (%)	26.8	22.5	18.8	20.8	19.5	21.5	23.2
Healthcare visits (SD)	2.1 (1.4)	2.1 (1.4)	2.1 (1.4)	2.2 (1.4)	2.1 (1.5)	2.1 (1.4)	2.1 (1.4)
Hospital overnight (SD)	0.2 (0.6)	0.2 (0.6)	0.2 (0.5)	0.2 (0.6)	0.2 (0.6)	0.2 (0.6)	0.2 (0.6)
**Race (ref. White)**							
Mexican	7512 (21.3)	1550 (28.9)	1299 (21.7)	1159 (20.7)	1185 (21.3)	1096 (17.6)	1223 (18.8)
Hispanic	2301 (6.5)	337 (6.2)	258 (4.3)	168 (3.0)	170 (3.1)	710 (11.4)	658 (10.1)
White	16 771 (47.5)	2329 (42.8)	3027 (50.6)	2857 (50.9)	2633 (47.4)	2855 (45.9)	3070 (47.2)
Black	7238 (20.5)	1034 (19.0)	1184 (19.8)	1186 (21.1)	1340 (24.1)	1300 (20.9)	1194 (18.3)
Other	1505 (4.3)	193 (3.6)	217 (3.6)	240 (4.3)	232 (4.2)	258 (4.2)	365 (5.6)

**Table 3.** Multivariable associations between demographic adjustments and all-cause mortality in NHANES 1999–2010.

	HR	95% CI	*P* value
Age (per 1 SD)	3.82	3.07, 4.75	<1 × 10^−10^
Age-squared (per 1 SD)	1.27	1.16, 1.40	6.09 × 10^−7^
Female	0.61	0.57, 0.66	<1 × 10^−10^
Income: poverty ratio (per 1 SD)	0.76	0.73, 0.79	<1 × 10^−10^
Healthcare visits (per 1 visit)	1.24	1.19, 1.30	<1 × 10^−10^
Overnight in hospital (per 1 visit)	1.12	1.09, 1.15	<1 × 10^−10^
Race (reference: White)			
Mexican American	0.78	0.70, 0.87	8.75 × 10^−6^
Other Hispanic	0.73	0.61, 0.88	1.20 × 10^−3^
African American	1.20	1.07, 1.33	1.18 × 10^−3^
Other race	0.79	0.57, 1.09	0.15
*N* (deceased)	35 327 (3663)		

**Table 4.** 20 biomarkers associated with mortality in the US. SD represents the standard deviation for the distribution of values and the unit change reflected by the HR for mortality. [10, 90] represent the lower 10th and upper 90th percentiles of the original population distribution considered for this analysis

Measure	HR (per SD)	CI	SD	[10, 90]	*I*^2^	*n*	*q* value
Trochanter BMC	0.708	0.591, 0.847	1.89	5.49, 10.72	0	3	1.04 × 10^−2^
Weight (kg)	0.722	0.608, 0.858	22.4	23.8, 88.5	49.8	6	1.21 × 10^−2^
Intertrochanter BMD	0.753	0.643, 0.881	0.14	0.914, 1.301	0	3	2.03 × 10^−2^
Albumin (g / dL)	0.798	0.752, 0.848	0.25	3.9, 4.6	0	6	8.51 × 10^−11^
Subscapular skinfold (mm)	0.821	0.763, 0.883	6.53	6.8, 24.4	0	6	2.58 × 10^−5^
Maximal calf circumference (cm)	0.848	0.788, 0.912	3.09	32.7, 41.2	0	4	8.95 × 10^−4^
Body Mass Index (kg / m^2^)	0.866	0.804, 0.931	4.88	17.96, 31.53	0.0145	6	8.37 × 10^−3^
Lumber pelvis BMD (g / cm^2^)	0.882	0.821, 0.947	0.14	1.027, 1.43	0.00638	4	2.55 × 10^−2^
Transferrin saturation (%)	0.882	0.82, 0.948	6.82	13.1, 31.8	0.0561	4	2.78 × 10^−2^
Iron from refrigerated serum (μg / dL)	0.898	0.847, 0.951	22.9	53, 116	0.00535	5	1.25 × 10^−2^
Thigh circumference (cm)	0.901	0.847, 0.957	4.89	44.5, 58	0	4	2.78 × 10^−2^
Sodium (mmol / L)	0.901	0.855, 0.949	1.62	137, 141	0	5	8.37 × 10^−3^
60 second pulse	1.13	1.06, 1.2	8.73	62, 86	0	6	8.50 × 10^−3^
C—reactive protein (mg / dL)	1.13	1.07, 1.18	0.20	0.01, 0.49	0.00423	6	3.32 × 10^−4^
White blood cell count (1000 cells/ μL)	1.13	1.06, 1.2	1.40	5.3, 9.1	0	6	8.37 × 10^−3^
Segmented neutrophils number	1.17	1.1, 1.25	1.09	2.5, 5.5	13	6	4.99 × 10^−5^
Globulin (g / dL)	1.18	1.07, 1.3	0.30	2.5, 3.4	53.2	5	2.53 × 10^−2^
Albumin, urine (μg/ mL)	1.22	1.15, 1.29	8.98	3.5, 24.7	24.5	6	2.59 × 10^−8^
Red cell distribution width (%)	1.23	1.17, 1.31	0.53	12, 13.4	0	6	5.77 × 10^−11^
Alkaline phosphatase (U / L)	1.23	1.09, 1.38	20.29	54, 107	80.4	6	2.68 × 10^−2^

*I*^2^ is a measure of heterogeneity in the meta-analysis between cohort survey years. *n* represents how many survey years this biomarker was observed.
