# Supplementary material for: Association of 152 Biomarker Reference Intervals with All-Cause Mortality in Participants of a General United States Survey from 1999 to 2010
Source: Clin Chem. Author manuscript; Available in PMC 2021 May 24. (PMC8142683; doi:10.1093/clinchem/hvaa271)
Supplement: Supplementary Information [file NIHMS1693134-supplement-Supplementary_Information.docx]

##### **Figure S1**. Bland-Altman plot for a sensitivity study of using different data cutoffs, mean of association sizes versus difference in association sizes. Limits of two standard deviations from the mean.

[ REF: FigS1-ba.tif ]

##### **Figure S2**. Pairwise Pearson correlations between all 152 biomarkers.

[ REF: FigS2-heat-all.tif ]

##### **Figure S3**. Heatmap of pairwise Pearson correlations among the 20 biomarkers associated with mortality.

[ REF: FigS3-heat-sig.tif ]

#####

##### **Table S1**. List of 152 biomarkers used in the study.

|  | **NHANES name** | **Description** | **Category** | **Surveys** |
| --- | --- | --- | --- | --- |
| **1** | BAXFTC11 | cond 1 trial 1 failure time, cond 1 trial 1 failure time | physical functioning | 1999-2000, 2001-2002, 2003-2004 |
| **2** | BAXFTC12 | cond 1 trial 2 failure time, cond 1 trial 2 failure time | physical functioning | 1999-2000, 2001-2002, 2003-2004 |
| **3** | BAXFTC21 | cond 2 trial 1 failure time, cond 2 trial 1 failure time | physical functioning | 1999-2000, 2001-2002, 2003-2004 |
| **4** | BAXFTC22 | cond 2 trial 2 failure time, cond 2 trial 2 failure time | physical functioning | 1999-2000, 2001-2002, 2003-2004 |
| **5** | BAXFTC31 | cond 3 trial 1 failure time, cond 3 trial 1 failure time | physical functioning | 1999-2000, 2001-2002, 2003-2004 |
| **6** | BAXFTC32 | cond 3 trial 2 failure time, cond 3 trial 2 failure time | physical functioning | 1999-2000, 2001-2002, 2003-2004 |
| **7** | BAXFTC41 | cond 4 trial 1 failure time, cond 4 trial 1 failure time | physical functioning | 1999-2000, 2001-2002, 2003-2004 |
| **8** | BAXFTC42 | cond 4 trial 2 failure time, cond 4 trial 2 failure time | physical functioning | 1999-2000, 2001-2002, 2003-2004 |
| **9** | BAXPFC11 | cond 1 trial 1 feet together eyes open | physical functioning | 1999-2000, 2001-2002, 2003-2004 |
| **10** | BAXPFC12 | cond 1 trial 2 feet together eyes open | physical functioning | 1999-2000, 2001-2002, 2003-2004 |
| **11** | BAXPFC21 | cond 2 trial 1 feet together eyes closed | physical functioning | 1999-2000, 2001-2002, 2003-2004 |
| **12** | BAXPFC22 | cond 2 trial 2 feet together eyes closed | physical functioning | 1999-2000, 2001-2002, 2003-2004 |
| **13** | BAXPFC31 | cond 3 trial 1 foam pad eyes open | physical functioning | 1999-2000, 2001-2002, 2003-2004 |
| **14** | BAXPFC32 | cond 3 trial 2 foam pad eyes open | physical functioning | 1999-2000, 2001-2002, 2003-2004 |
| **15** | BAXPFC41 | cond 4 trial 1 foam pad eyes closed | physical functioning | 1999-2000, 2001-2002, 2003-2004 |
| **16** | BAXPFC42 | cond 4 trial 2 foam pad eyes closed | physical functioning | 1999-2000, 2001-2002, 2003-2004 |
| **17** | BMXBMI | body mass index | body measures | 1999-2000, 2001-2002, 2003-2004, 2005-2006, 2007-2008, 2009-2010, 2011-2012, 2013-2014 |
| **18** | BMXCALF | maximal calf circumference | body measures | 1999-2000, 2001-2002, 2003-2004, 2005-2006 |
| **19** | BMXHEAD | head circumference | body measures | 1999-2000, 2001-2002, 2003-2004, 2005-2006, 2007-2008, 2009-2010, 2011-2012, 2013-2014 |
| **20** | BMXHT | standing height | body measures | 1999-2000, 2001-2002, 2003-2004, 2005-2006, 2007-2008, 2009-2010, 2011-2012, 2013-2014 |
| **21** | BMXLEG | upper leg length | body measures | 1999-2000, 2001-2002, 2003-2004, 2005-2006, 2007-2008, 2009-2010, 2011-2012, 2013-2014 |
| **22** | BMXRECUM | recumbent length | body measures | 1999-2000, 2001-2002, 2003-2004, 2005-2006, 2007-2008, 2009-2010, 2011-2012, 2013-2014 |
| **23** | BMXSUB | subscapular skinfold | body measures | 1999-2000, 2001-2002, 2003-2004, 2005-2006, 2007-2008, 2009-2010 |
| **24** | BMXTHICR | thigh circumference | body measures | 1999-2000, 2001-2002, 2003-2004, 2005-2006 |
| **25** | BMXTRI | triceps skinfold | body measures | 1999-2000, 2001-2002, 2003-2004, 2005-2006, 2007-2008, 2009-2010 |
| **26** | BMXWAIST | waist circumference | body measures | 1999-2000, 2001-2002, 2003-2004, 2005-2006, 2007-2008, 2009-2010, 2011-2012, 2013-2014 |
| **27** | BMXWT | weight | body measures | 1999-2000, 2001-2002, 2003-2004, 2005-2006, 2007-2008, 2009-2010, 2011-2012, 2013-2014 |
| **28** | BPXCHR | 60 sec heart rate | blood pressure | 1999-2000, 2001-2002, 2003-2004, 2005-2006, 2007-2008, 2009-2010, 2011-2012, 2013-2014 |
| **29** | BPXPLS | 60 sec pulse | blood pressure | 1999-2000, 2001-2002, 2003-2004, 2005-2006, 2007-2008, 2009-2010, 2011-2012, 2013-2014 |
| **30** | CVDESVO2 | estimated vo2max | physical fitness | 1999-2000, 2001-2002, 2003-2004 |
| **31** | CVDR1DI | recovery 1 diastolic bp | physical fitness | 1999-2000, 2001-2002, 2003-2004 |
| **32** | CVDR1HR | recovery 1 heart rate | physical fitness | 1999-2000, 2001-2002, 2003-2004 |
| **33** | CVDR1SY | recovery 1 systolic bp | physical fitness | 1999-2000, 2001-2002, 2003-2004 |
| **34** | CVDR2DI | recovery 2 diastolic bp | physical fitness | 1999-2000, 2001-2002, 2003-2004 |
| **35** | CVDR2HR | recovery 2 heart rate | physical fitness | 1999-2000, 2001-2002, 2003-2004 |
| **36** | CVDR2SY | recovery 2 systolic bp | physical fitness | 1999-2000, 2001-2002, 2003-2004 |
| **37** | CVDS1DI | stage 1 diastolic bp | physical fitness | 1999-2000, 2001-2002, 2003-2004 |
| **38** | CVDS1HR | stage 1 heart rate | physical fitness | 1999-2000, 2001-2002, 2003-2004 |
| **39** | CVDS1SY | stage 1 systolic bp | physical fitness | 1999-2000, 2001-2002, 2003-2004 |
| **40** | CVDS2DI | stage 2 diastolic bp | physical fitness | 1999-2000, 2001-2002, 2003-2004 |
| **41** | CVDS2HR | stage 2 heart rate | physical fitness | 1999-2000, 2001-2002, 2003-2004 |
| **42** | CVDS2SY | stage 2 systolic bp | physical fitness | 1999-2000, 2001-2002, 2003-2004 |
| **43** | CVDVOMAX | predicted vo2max | physical fitness | 1999-2000, 2001-2002, 2003-2004 |
| **44** | DXXFMBCC | total femur bmd invalidity code | body measures | 2005-2006, 2007-2008, 2009-2010, 2013-2014 |
| **45** | DXXHEBMD | head bmd | body measures | 1999-2000, 2001-2002, 2003-2004, 2005-2006 |
| **46** | DXXINA | intertrochanter area | body measures | 2005-2006, 2007-2008, 2009-2010, 2013-2014 |
| **47** | DXXINBMC | intertrochanter bone mineral content | body measures | 2005-2006, 2007-2008, 2009-2010, 2013-2014 |
| **48** | DXXINBMD | intertrochanter bone mineral density | body measures | 2005-2006, 2007-2008, 2009-2010, 2013-2014 |
| **49** | DXXL1A | l1 area | body measures | 2005-2006, 2007-2008, 2009-2010, 2013-2014 |
| **50** | DXXL1BCC | l1 bmd invalidity code | body measures | 2005-2006, 2007-2008, 2009-2010, 2013-2014 |
| **51** | DXXL1BMC | l1 bone mineral composition | body measures | 2005-2006, 2007-2008, 2009-2010, 2013-2014 |
| **52** | DXXL1BMD | l1 bone mineral density | body measures | 2005-2006, 2007-2008, 2009-2010, 2013-2014 |
| **53** | DXXL2A | l2 area | body measures | 2005-2006, 2007-2008, 2009-2010, 2013-2014 |
| **54** | DXXL2BCC | l2 bmd invalidity code | body measures | 2005-2006, 2007-2008, 2009-2010, 2013-2014 |
| **55** | DXXL2BMC | l2 bone mineral composition | body measures | 2005-2006, 2007-2008, 2009-2010, 2013-2014 |
| **56** | DXXL2BMD | l2 bone mineral density | body measures | 2005-2006, 2007-2008, 2009-2010, 2013-2014 |
| **57** | DXXL3A | l3 area | body measures | 2005-2006, 2007-2008, 2009-2010, 2013-2014 |
| **58** | DXXL3BCC | l3 bmd invalidity code | body measures | 2005-2006, 2007-2008, 2009-2010, 2013-2014 |
| **59** | DXXL3BMC | l3 bone mineral composition | body measures | 2005-2006, 2007-2008, 2009-2010, 2013-2014 |
| **60** | DXXL3BMD | l3 bone mineral density | body measures | 2005-2006, 2007-2008, 2009-2010, 2013-2014 |
| **61** | DXXL4A | l4 area | body measures | 2005-2006, 2007-2008, 2009-2010, 2013-2014 |
| **62** | DXXL4BCC | l4 bmd invalidity code | body measures | 2005-2006, 2007-2008, 2009-2010, 2013-2014 |
| **63** | DXXL4BMC | l4 bone mineral composition | body measures | 2005-2006, 2007-2008, 2009-2010, 2013-2014 |
| **64** | DXXL4BMD | l4 bone mineral density | body measures | 2005-2006, 2007-2008, 2009-2010, 2013-2014 |
| **65** | DXXLSBMD | lumber spine bmd | body measures | 1999-2000, 2001-2002, 2003-2004, 2005-2006 |
| **66** | DXXNKA | femoral neck area | body measures | 2005-2006, 2007-2008, 2009-2010, 2013-2014 |
| **67** | DXXNKBMC | femoral neck bone mineral content | body measures | 2005-2006, 2007-2008, 2009-2010, 2013-2014 |
| **68** | DXXNKBMD | femoral neck bone mineral density | body measures | 2005-2006, 2007-2008, 2009-2010, 2013-2014 |
| **69** | DXXOFA | total femur area | body measures | 2005-2006, 2007-2008, 2009-2010, 2013-2014 |
| **70** | DXXOFBMC | total femur bone mineral content | body measures | 2005-2006, 2007-2008, 2009-2010, 2013-2014 |
| **71** | DXXOFBMD | total femur bone mineral density | body measures | 2005-2006, 2007-2008, 2009-2010, 2013-2014 |
| **72** | DXXOSA | total spine area | body measures | 2005-2006, 2007-2008, 2009-2010, 2013-2014 |
| **73** | DXXOSBCC | total spine bmd invalidity code | body measures | 2005-2006, 2007-2008, 2009-2010, 2013-2014 |
| **74** | DXXOSBMC | total spine bmc | body measures | 2005-2006, 2007-2008, 2009-2010, 2013-2014 |
| **75** | DXXOSBMD | total spine bmd | body measures | 2005-2006, 2007-2008, 2009-2010, 2013-2014 |
| **76** | DXXPEBMD | lumber pelvis bmd | body measures | 1999-2000, 2001-2002, 2003-2004, 2005-2006 |
| **77** | DXXTRA | trochanter area | body measures | 2005-2006, 2007-2008, 2009-2010, 2013-2014 |
| **78** | DXXTRBMC | trochanter bone mineral content | body measures | 2005-2006, 2007-2008, 2009-2010, 2013-2014 |
| **79** | DXXTRBMD | trochanter bone mineral density | body measures | 2005-2006, 2007-2008, 2009-2010, 2013-2014 |
| **80** | DXXTRFAT | trunk fat | body measures | 1999-2000, 2001-2002, 2003-2004, 2005-2006 |
| **81** | DXXWDA | ward's triangle area | body measures | 2005-2006, 2007-2008, 2009-2010, 2013-2014 |
| **82** | DXXWDBMC | ward's triangle bone mineral content | body measures | 2005-2006, 2007-2008, 2009-2010, 2013-2014 |
| **83** | DXXWDBMD | ward's triangle bone mineral density | body measures | 2005-2006, 2007-2008, 2009-2010, 2013-2014 |
| **84** | LBDBANO | basophils number, basophils number | blood | 1999-2000, 2001-2002, 2003-2004, 2005-2006, 2007-2008, 2009-2010, 2011-2012 |
| **85** | LBDEONO | eosinophils number, eosinophils number | blood | 1999-2000, 2001-2002, 2003-2004, 2005-2006, 2007-2008, 2009-2010, 2011-2012 |
| **86** | LBDHDD | direct hdl-cholesterol | biochemistry | 2003-2004, 2005-2006, 2007-2008, 2009-2010, 2011-2012, 2013-2014 |
| **87** | LBDHDL | hdl-cholesterol | biochemistry | 1999-2000, 2001-2002, 2003-2004 |
| **88** | LBDLDL | ldl-cholesterol | biochemistry | 1999-2000, 2001-2002, 2003-2004, 2005-2006, 2007-2008, 2009-2010, 2011-2012 |
| **89** | LBDLYMNO | lymphocyte number | blood | 1999-2000, 2001-2002, 2003-2004, 2005-2006, 2007-2008, 2009-2010, 2011-2012 |
| **90** | LBDMONO | monocyte number | blood | 1999-2000, 2001-2002, 2003-2004, 2005-2006, 2007-2008, 2009-2010, 2011-2012 |
| **91** | LBDNENO | segmented neutrophils number | blood | 1999-2000, 2001-2002, 2003-2004, 2005-2006, 2007-2008, 2009-2010, 2011-2012 |
| **92** | LBDP3 | prostate specific antigen ratio | biochemistry | 2001-2002, 2003-2004, 2005-2006, 2007-2008, 2009-2010 |
| **93** | LBDPCT | transferrin saturation | biochemistry | 1999-2000, 2001-2002, 2003-2004, 2005-2006 |
| **94** | LBXAPB | apolipoprotein | biochemistry | 2005-2006, 2007-2008, 2009-2010, 2011-2012 |
| **95** | LBXBAP | bone alkaline phosphotase | biochemistry | 1999-2000, 2001-2002, 2003-2004 |
| **96** | LBXBAPCT | basophils percent | blood | 1999-2000, 2001-2002, 2003-2004, 2005-2006, 2007-2008, 2009-2010, 2011-2012 |
| **97** | LBXCPSI | c-peptide | biochemistry | 1999-2000, 2001-2002, 2003-2004 |
| **98** | LBXCRP | c-reactive protein | biochemistry | 1999-2000, 2001-2002, 2003-2004, 2005-2006, 2007-2008, 2009-2010 |
| **99** | LBXEOPCT | eosinophils percent | blood | 1999-2000, 2001-2002, 2003-2004, 2005-2006, 2007-2008, 2009-2010, 2011-2012 |
| **100** | LBXEPP | protoporphyrin | biochemistry | 1999-2000, 2001-2002, 2003-2004, 2005-2006 |
| **101** | LBXFER | ferritin | biochemistry | 1999-2000, 2001-2002, 2003-2004, 2005-2006, 2007-2008, 2009-2010 |
| **102** | LBXGH | glycohemoglobin | biochemistry | 1999-2000, 2001-2002, 2003-2004, 2005-2006, 2007-2008, 2009-2010, 2011-2012, 2013-2014 |
| **103** | LBXGLT | two hour glucose | biochemistry | 2005-2006, 2007-2008, 2009-2010, 2011-2012, 2013-2014 |
| **104** | LBXGLU | plasma glucose | biochemistry | 1999-2000, 2001-2002, 2003-2004, 2005-2006, 2007-2008, 2009-2010, 2011-2012, 2013-2014 |
| **105** | LBXHCT | hematocrit | blood | 1999-2000, 2001-2002, 2003-2004, 2005-2006, 2007-2008, 2009-2010, 2011-2012 |
| **106** | LBXHCY | homocysteine | biochemistry | 1999-2000, 2001-2002, 2003-2004, 2005-2006 |
| **107** | LBXHGB | hemoglobin | blood | 1999-2000, 2001-2002, 2003-2004, 2005-2006, 2007-2008, 2009-2010, 2011-2012 |
| **108** | LBXIN | insulin | hormone, biochemistry | 1999-2000, 2001-2002, 2003-2004, 2005-2006, 2007-2008, 2009-2010, 2011-2012 |
| **109** | LBXLYPCT | lymphocyte percent | blood | 1999-2000, 2001-2002, 2003-2004, 2005-2006, 2007-2008, 2009-2010, 2011-2012 |
| **110** | LBXMC | mchc | blood | 1999-2000, 2001-2002, 2003-2004, 2005-2006, 2007-2008, 2009-2010, 2011-2012 |
| **111** | LBXMCHSI | mean cell hemoglobin | blood | 1999-2000, 2001-2002, 2003-2004, 2005-2006, 2007-2008, 2009-2010, 2011-2012 |
| **112** | LBXMCVSI | mean cell volume | blood | 1999-2000, 2001-2002, 2003-2004, 2005-2006, 2007-2008, 2009-2010, 2011-2012 |
| **113** | LBXMMA | methylmalonic acid | biochemistry | 1999-2000, 2001-2002, 2003-2004 |
| **114** | LBXMOPCT | monocyte percent | blood | 1999-2000, 2001-2002, 2003-2004, 2005-2006, 2007-2008, 2009-2010, 2011-2012 |
| **115** | LBXMPSI | mean platelet volume | blood | 1999-2000, 2001-2002, 2003-2004, 2005-2006, 2007-2008, 2009-2010, 2011-2012 |
| **116** | LBXNEPCT | segmented neutrophils percent | blood | 1999-2000, 2001-2002, 2003-2004, 2005-2006, 2007-2008, 2009-2010, 2011-2012 |
| **117** | LBXP1 | total prostate specific antigen | biochemistry | 2001-2002, 2003-2004, 2005-2006, 2007-2008, 2009-2010 |
| **118** | LBXP2 | free prostate specific antigen | biochemistry | 2001-2002, 2003-2004, 2005-2006, 2007-2008, 2009-2010 |
| **119** | LBXPLTSI | platelet count | blood | 1999-2000, 2001-2002, 2003-2004, 2005-2006, 2007-2008, 2009-2010, 2011-2012 |
| **120** | LBXRBCSI | red cell count | blood | 1999-2000, 2001-2002, 2003-2004, 2005-2006, 2007-2008, 2009-2010, 2011-2012 |
| **121** | LBXRDW | red cell distribution width | blood | 1999-2000, 2001-2002, 2003-2004, 2005-2006, 2007-2008, 2009-2010, 2011-2012 |
| **122** | LBXSAL | albumin | biochemistry | 1999-2000, 2001-2002, 2003-2004, 2005-2006, 2007-2008, 2009-2010, 2011-2012, 2013-2014 |
| **123** | LBXSAPSI | alkaline phosphatase | biochemistry | 1999-2000, 2001-2002, 2003-2004, 2005-2006, 2007-2008, 2009-2010, 2011-2012, 2013-2014 |
| **124** | LBXSASSI | asparate aminotransferase | biochemistry | 1999-2000, 2001-2002, 2003-2004, 2005-2006, 2007-2008, 2009-2010, 2011-2012, 2013-2014 |
| **125** | LBXSATSI | alanine aminotransferase | biochemistry | 1999-2000, 2001-2002, 2003-2004, 2005-2006, 2007-2008, 2009-2010, 2011-2012, 2013-2014 |
| **126** | LBXSBU | blood urea nitrogen | biochemistry | 1999-2000, 2001-2002, 2003-2004, 2005-2006, 2007-2008, 2009-2010, 2011-2012, 2013-2014 |
| **127** | LBXSC3SI | bicarbonate | biochemistry | 1999-2000, 2001-2002, 2003-2004, 2005-2006, 2007-2008, 2009-2010, 2011-2012, 2013-2014 |
| **128** | LBXSCA | total calcium | biochemistry | 1999-2000, 2001-2002, 2003-2004, 2005-2006, 2007-2008, 2009-2010, 2011-2012, 2013-2014 |
| **129** | LBXSCH | total cholesterol | biochemistry | 1999-2000, 2001-2002, 2003-2004, 2005-2006, 2007-2008, 2009-2010, 2011-2012, 2013-2014 |
| **130** | LBXSCLSI | chloride | biochemistry | 1999-2000, 2001-2002, 2003-2004, 2005-2006, 2009-2010, 2011-2012, 2013-2014 |
| **131** | LBXSCR | urine creatinine | biochemistry | 1999-2000, 2001-2002, 2003-2004, 2005-2006, 2007-2008, 2009-2010, 2011-2012, 2013-2014 |
| **132** | LBXSGB | globulin | biochemistry | 1999-2000, 2001-2002, 2003-2004, 2005-2006, 2009-2010, 2011-2012, 2013-2014 |
| **133** | LBXSGL | serum glucose | biochemistry | 1999-2000, 2001-2002, 2003-2004, 2005-2006, 2009-2010, 2011-2012, 2013-2014 |
| **134** | LBXSGTSI | gamma glutamyl transferase | biochemistry | 1999-2000, 2001-2002, 2003-2004, 2005-2006, 2009-2010, 2011-2012, 2013-2014 |
| **135** | LBXSIR | iron | biochemistry | 1999-2000, 2001-2002, 2003-2004, 2005-2006, 2009-2010, 2011-2012, 2013-2014 |
| **136** | LBXSKSI | potassium | biochemistry | 1999-2000, 2001-2002, 2003-2004, 2005-2006, 2009-2010, 2011-2012, 2013-2014 |
| **137** | LBXSLDSI | lactate dehydrogenase | biochemistry, hormone | 1999-2000, 2001-2002, 2003-2004, 2005-2006, 2009-2010, 2011-2012, 2013-2014 |
| **138** | LBXSNASI | sodium | biochemistry | 1999-2000, 2001-2002, 2003-2004, 2005-2006, 2009-2010, 2011-2012, 2013-2014 |
| **139** | LBXSOSSI | osmolality | biochemistry | 1999-2000, 2001-2002, 2003-2004, 2005-2006, 2009-2010, 2011-2012, 2013-2014 |
| **140** | LBXSPH | phosphorus | biochemistry, nutrients | 1999-2000, 2001-2002, 2003-2004, 2005-2006, 2009-2010, 2011-2012, 2013-2014 |
| **141** | LBXSTB | total bilirubin | biochemistry | 1999-2000, 2001-2002, 2003-2004, 2005-2006, 2009-2010, 2011-2012, 2013-2014 |
| **142** | LBXSTP | total protein | biochemistry | 1999-2000, 2001-2002, 2003-2004, 2005-2006, 2009-2010, 2011-2012, 2013-2014 |
| **143** | LBXSUA | uric acid | biochemistry | 1999-2000, 2001-2002, 2003-2004, 2005-2006, 2009-2010, 2011-2012, 2013-2014 |
| **144** | LBXTC | total cholesterol | biochemistry | 1999-2000, 2001-2002, 2003-2004, 2005-2006, 2007-2008, 2009-2010, 2011-2012, 2013-2014 |
| **145** | LBXTFR | transferrin receptor | biochemistry | 2003-2004, 2005-2006, 2007-2008, 2009-2010 |
| **146** | LBXTIB | total iron binding capacity | biochemistry | 1999-2000, 2001-2002, 2003-2004, 2005-2006 |
| **147** | LBXTR | triglyceride | biochemistry | 1999-2000, 2001-2002, 2003-2004, 2005-2006, 2007-2008, 2009-2010, 2011-2012 |
| **148** | LBXWBCSI | white blood cell count | blood | 1999-2000, 2001-2002, 2003-2004, 2005-2006, 2007-2008, 2009-2010, 2011-2012 |
| **149** | MDIS | mean diastolic | blood pressure | 1999-2000, 2001-2002, 2003-2004, 2005-2006 |
| **150** | MSYS | mean systolic | blood pressure | 1999-2000, 2001-2002, 2003-2004, 2005-2006 |
| **151** | URXUCR | urine creatinine | biochemistry, phenols, diakyl, hydrocarbons, perchlorate, phthalates, pesticides, heavy metals, nutrients | 1999-2000, 2001-2002, 2003-2004, 2005-2006, 2007-2008, 2009-2010, 2011-2012, 2013-2014 |
| **152** | URXUMA | urine albumin | biochemistry | 1999-2000, 2001-2002, 2003-2004, 2005-2006, 2007-2008, 2009-2010, 2011-2012, 2013-2014 |
