# Supplementary figures and images for: Association of 152 Biomarker Reference Intervals with All-Cause Mortality in Participants of a General United States Survey from 1999 to 2010
Source: Clin Chem. Author manuscript; Available in PMC 2021 May 24. (PMC8142683; doi:10.1093/clinchem/hvaa271)

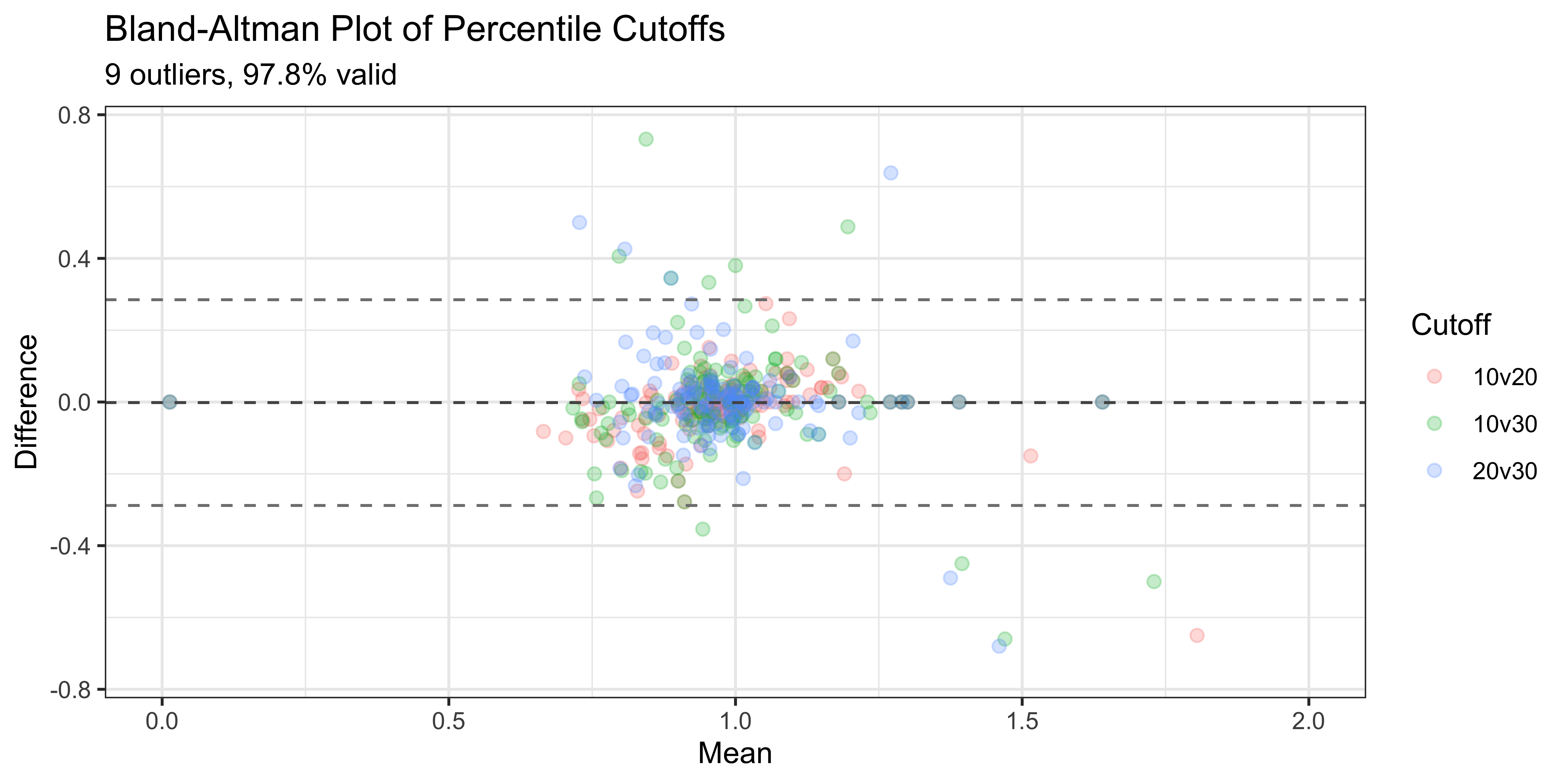

Supplement: Fig S1 [file NIHMS1693134-supplement-Fig_S1.tif]
